# Semi-Continuous Desalination and Concentration of Small-Volume Samples

**DOI:** 10.3390/ijms222312904

**Published:** 2021-11-29

**Authors:** David Tichý, Zdeněk Slouka

**Affiliations:** 1 Department of Chemical Engineering, University of Chemistry and Technology Prague, Technická 3, 16628 Prague, Czech Republic; tichyd@vscht.cz; 2 New Technologies—Research Centre, University of West Bohemia, Univerzitní 8, 30614 Plzeň, Czech Republic

**Keywords:** desalination, cation-exchange membranes, ion concentration polarization, ion separation, microfluidics

## Abstract

Electrodialysis is an electric-field-mediated process separating ions exploiting selective properties of ion-exchange membranes. The ion-exchange membranes create an ion-depleted zone in an electrolyte solution adjacent to the membrane under DC polarization. We constructed a microfluidic system that uses the ion-depleted zone to separate ions from the processed water solution. We tested the separation performance by desalting a model KCl solution spiked with fluorescein for direct observation. We showed both visually and by measuring the conductivity of the output solutions that the system can work in three modes of operation referred to as continuous desalination, desalination by accumulation, and unsuccessful desalination. The mode of operation can easily be set by changing the control parameters. The desalination factors for the model KCl solution reached values from 80 to 100%, depending on the mode of operation. The concentration factor, given as a ratio of concentrate-to-feed concentrations, reached zero for desalination by accumulation when only diluate was produced. The water recovery, therefore, was infinite at these conditions. Independent control of the diluate and concentrate flow rates and the DC voltage turned our system into a versatile platform, enabling us to set proper conditions to process various samples.

## 1. Introduction

Ion-exchange membranes (IEMs) [1,2] are widely used in (electro-)membrane separation processes [3] for the removal of small solutes possessing charge under given conditions. These separation processes include electrodialysis (ED) [4], electrodeionization (EDI) [5], bipolar membrane electrodialysis (BMED) [6], capacitive deionization (CDI) [7], or diffusion dialysis (DD) [8]. Besides the field of ionic separations, processes providing energy generation, such as reverse electrodialysis (RED) [9], fuel cells (FCs) [10], and redox flow batteries (RFBs) [11], also employ IEMs. In all the mentioned cases, selective transport of charged solutes, dictated by a property called ion selectivity [12], occurs in the membranes. The ion selectivity is caused by a so-called fixed charge bound on/in the membrane. The fixed charge, given mainly by dissociated functional groups, interacts electrostatically with mobile ionic species in the electrolyte and ensures preferential permeation of counterions through the membrane. However, ion selectivity also affects the processes proceeding in the electrolyte solutions adjacent to the membrane, especially when under DC polarization. Recently, a new group of techniques based on these processes has appeared and gained interest in the scientific community [13]. The term ion concentration polarization (ICP) was coined and accepted. ICP has a rather broad meaning and, among others, includes the behavior of ion-selective systems in the DC electric field in association with preconcentration or removal of charged entities [14].

ICP is a phenomenon that occurs on the connection of a DC electric field to an ion-selective material represented by, for example, an ion-exchange membrane, ion-exchange resin particle, or ion-selective nanochannel. The simultaneous effect of an external electric field and ion selectivity results in the selective and directed transport of ionic species accompanied by concentration polarization of the ion-selective materials. Specifically, the concentration polarization depletes ions from the electrolyte solution on one side of such a system and enriches them on the other one [15,16]. The electrolyte solution zones adjacent to the system are referred to as ion-depleted and ion-concentrated zones, respectively. When extended far into the electrolyte solution, the ion-depleted zone is a powerful tool to control the transport of ionic entities in the electrolyte bulk leading to local preconcentration or deionization of the flowing liquid. The ion-depleted zone can, thus, be viewed as a nonmechanical ionic filter [17]. The concentration polarization and generation of ion-depleted and concentrated zones are strongly affected by overlimiting phenomena pertinent to ion-selective systems [18]. Electroconvection and water-splitting are considered the most important [19,20]. Electroconvention denotes the convective motion of the electrolyte solution invoked by a strong electric field acting on a nonequilibrium space charge localized at the polarized membrane surface [21]. The electroconvection usually manifests itself as an array of vortices mixing the ion-depleted zone with the electrolyte solution bulk if available [22]. The mixing leads to the ion concentration increase in the ion-depleted zone, which, in turn, leads to the appearance of the overlimiting current. The overlimiting current is a current larger than that theoretically predicted from the concentration polarization theory. Water-splitting was shown to occur at some ion-exchange membranes and resin particles when overlimiting current passes through the system [23,24]. Water-splitting localizes to the membrane-electrolyte solution interface and generates new ion-current carriers, namely H^+^ ad OH^−^ ions. The possibility to reach an overlimiting current makes the traditional IEM-based separations unique. Other membrane separations (e.g., pressure-driven operations) are limited by concentration polarization, determining the maximum possible flux through the membrane [25]. 

The mechanisms underlying the appearance of the ion-depleted zone and the overlimiting phenomena significantly depend on the system geometry and experimental conditions. For example, the ion-depleted zone spanning the cross-section of a microfluidic channel has been used for the removal (preconcentration) of all ionic species from flowing water solutions. The ion-preconcentration efficiency was controlled by the velocity of the flow in the channel and the applied voltage sustaining the ion-depleted zone [26]. The stronger the electric force when being used to develop the ion-depleted zone, the better its stability after applying convective flow in the channel. We showed that tuning the strength of the electric field and the flow rate in the channel also predefines the length of the ion-depleted zone and the position of the ion-preconcentrated band. 

The ICP phenomenon has been exploited in many novel technologies, such as preconcentration [27], biosensing [28], separation, ionic control [29], and desalination [30]. For instance, the electric voltage imposed on a passive Nafion membrane strip in a microfluidic device preconcentrated a model protein [31], and lipid vesicles [32]. The voltage polarized the strip producing the ion-depleted and ion-concentrated zones within the same channel, and invoked the electroosmotic flow to deliver the ions for preconcentration (EOF). The simultaneous action of the ion-depleted zone and EOF led to the accumulation of ions and ionic entities at the ion-depleted front edge. A similar approach was used to preconcentrate blood-based biofluids in a paper-based preconcentrator [33]. Two cation-exchange membranes (marked as concentrating and depleting) connected in series can also produce ion-depleted and ion-concentrated zones in a single channel [34]. In this case, the electrolyte regions with significant differences in ionic concentration generate a strongly nonlinear profile of the electric field in the channel, yielding a spatially dependent electric force. The preconcentration of charged molecules then occurs between the two membranes due to the nonlinear electric force profile. The position of the band preconcentrated molecules followed by tracking real-time fluorescence depended on the connected electric voltage, volumetric flow rate of the solution through the channels, and channel geometry. The preconcentration of large charge molecules was accompanied by removing small ions from the electrolyte solution, leading to desalination. These ions accumulate similarly to the tracked fluorescently labeled molecules between the two cation exchange membranes. This microfluidic chip thus operates in a semi-batch mode.

Besides the analyte preconcentration, the separation of inorganic salts or water desalination are other examples of successful ICP-based applications. Water desalination aims to produce potable water from brackish or seawaters. The research in this field focuses on developing new technologies or the improvement of existing ones. Directional solvent extraction [35] or absorption into a graphene oxide-embedded paper matrix [36] are examples of new desalination processes. Integrating IEMs into capacitive deionization units is an example of improving the existing technologies. In capacitive deionization, the membranes, thanks to their intrinsic ion selectivity, show a positive effect on the efficiency of the process [37]. Another example of this approach is the evaluation of the benefits of operating the electrodialysis units in the overlimiting regime [18]. The mechanisms driving the overlimiting current enhance the mass transfer rate, which brings savings on the required amount of functional membranes, specifically on the area of those membranes. However, the application of the overlimiting regime is conditioned by connecting larger voltages, resulting in larger energy consumption. It can also be accompanied by unwanted phenomena having even more harmful effects on the units (e.g., water-splitting).

The first successful realization of ICP-based continuous desalination was achieved by Kim et al. [30]. The authors used an ion-selective nanojunction to produce an ion-depleted zone and remove small ions from the processed seawater. Two output streams were continuously withdrawn from the chip, the concentrated brine, and the desalted water. In a single-step operation, the salt removal efficiency reached 99% when 50% of the incoming seawater was recovered as the desalted water. The authors claimed that this desalting operation could not compete with large-scale desalination plants utilizing reverse osmosis, electrodialysis of distillation. Still, it can be helpful in small-scale applications. An improvement of the ICP-based desalination technique was suggested by the Ref. [14]. Their system contained two cation-exchange membranes producing ion-depleted and ion-concentrated zones and a porous membrane placed in between the two CEMs stabilizing the flow in the diluate and the concentrate channels. Such a geometry allowed counter-current arrangement of the diluate and concentrate streams. The authors employed flow rates yielding up to a 425-fold concentrated solution when processing 10 mM NaCl solutions.

The scale-up of ICP desalination is challenging due to the unstable nature of the ion-depleted zone. To tackle the ICP-based desalination scale-up challenge, MacDonald et al. employed ICP produced in a microfluidic chip with a nanoporous membrane [38]. The authors enlarged the desalination channel width to 2 cm and successfully increased its throughput while maintaining water desalination at around 90%. Another recent ICP-based desalination technique is shock electrodialysis [39]. Bazant et al. demonstrated a water purification system containing a porous frit placed between two CEMs. A deionization shock wave emerges when the electric current passes through the system, producing ion-depleted and ion-concentrated zones. The processed water then splits into two outlet streams. Knust et al. contributed to the ICP desalination research by utilizing faradaic ICP [40]. Their device uses an electrode instead of an ion-selective material and an electrochemical reaction to deplete the electrolyte solution of ions. In their case, the ion-depleted zone is formed at an anodic pole of the bipolar electrode.

Here, we design, fabricate, and characterize a novel microfluidic continuous ICP-based system for water desalination and charged species concentration with easily adjustable parameters to obtain the product streams (diluate and concentrate) of the required quality. A pair of pumps controls the flow rates in the diluate and concentrate channels. The flow rates are thus independent of the voltage applied to create the ion-depleted zone. The zone, produced at one of two in-series-connected cation exchange membranes, keeps ionic species in the main (concentrate) channel. We experimentally study the desalination efficiency and stability of preconcentration as a function of the volumetric flow rates and the applied voltage. We create an operational map of the constructed device by evaluating the obtained data, illustrating its versatile use for desalination and concentration.

## 2. Results

### 2.1. Determination of Outlet Concentrations

Before the desalination experiments, we measured the calibration curves to relate the measured root mean square (RMS) voltage (*U*_2_) and the concentration of the flowing electrolyte solution. First, the magnitude of the electric impedance denoted as *R*_2_ was calculated from the known RMS voltage *U*_2_ and the AC amplitude I_m_ (*R*_2_ = *U*_2_*/*(*I*_m_/√2)). This impedance value is proportional to the KCl solution concentration. The calibration was performed independently for both electrode pairs because minor differences in the geometry of the channel and the electrodes affect the measured data. We divided the range of KCl concentrations used for the calibration into two parts. The first one included the concentrations was from 0 to 0.2 mM, and the second from 0.2 mM to 20 mM. Since the measured impedance is indirectly proportional to the concentration, we fitted the measured data with polynomial functions of 1/*R*_2_. A quadratic function was used for the lower concentration range and linear relation for the other one. The developed correlations are given in Table 1 for both the concentrate and diluate channels.

### 2.2. Principle of Operation

**Figure 1:**
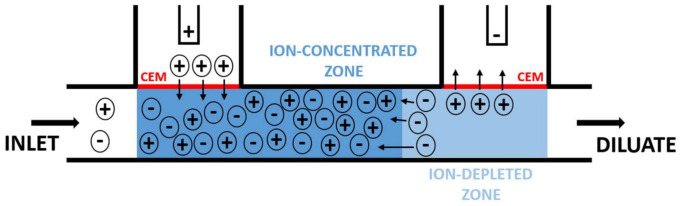
The principle of the chip operation: the passing current sustains the developed ion-depleted and ion-concentrated zones. The electric current is borne by ionic counterions, that is, cations in the case of cation-exchange membranes.

The principle of operation (see Figure 1) is based on the behavior of two in-series cation-exchange membranes integrated into a microfluidic system with an imposed DC electric field [26]. The in-series connection of the membranes results in the simultaneous creation of the ion-depleted and ion-concentrated zones in a single channel. The presence of those zones, in turn, indicates the generation of a concentration (thus, electrolytic conductivity) gradient along the channel, which causes the DC field to become highly nonlinear. It reaches tens of kV/m in the ion-depleted zone and only hundreds of V/m in the ion-concentrated zone [26]. The nonlinear profile in the electric field provides an electric force on ionic species that increases from the ion-concentrated zone to the ion-depleted zone. If acting against the applied convective flow, this force can efficiently separate charged large molecules (such as DNA in [26]). Since the system (except the interfaces) must obey the electroneutrality condition, the charged large molecules separate along with small ions, neutralizing their charge.

A strong local electric field sustains the ion-depleted zone. The field quickly removes membrane counterions entering the ion-depleted by pushing them through the depleting membrane. The membrane co-ions are virtually prohibited from entering this zone owing to the electric force pushing the co-ions away from this zone. The counterions migrating into the main channel through the auxiliary membrane preserve the electroneutrality condition, that is, no net charge due to the excluded co-ions occurs in the channel. This phenomenon accumulates small ions in front of the ion-depleted zone, similarly to the large counterions.

### 2.3. Types of Chip Operation

We conducted an experimental study in which the effect of three parameters was under scrutiny. These three parameters were the imposed voltage, the flow rate through the diluate channel, and the flow rate through the concentrate channel. The used combinations of the tested parameters are given in Section 3.4. While the imposed voltage controls the developed ion-depleted zone, the flow rates through the channels influence the amount of the recovered desalted water and the preconcentrated solution. The optimal conditions of the desalination should result in the production of a large amount of the desalted water and a small amount of the concentrate. The flow rate through the concentrate determines the theoretical maximum concentration in that channel. The microfluidic chip can thus be used for both desalination and preconcentration.

On performing the experimental study, we noticed that the desalination results could be divided into three distinct categories. The division was based on the desalination dynamics and the quality of the desalination and concentration. These three categories are denoted as (i) continuous desalination, (ii) desalination by accumulation, and (iii) unsuccessful operation.

**Figure 2:**
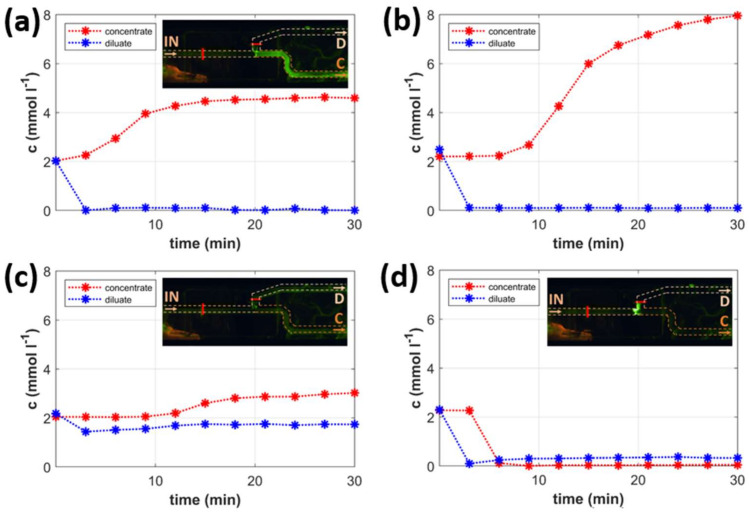
Types of chip operation: the graphs capture the measured time dependencies of the KCl concentrations in the diluate and concentrate stream, the insets the signal of fluorescein: (**a**) continuous desalination (*U* = 200 V, *V*_D_ = 20 μL/min, *V*_C_ = 5 μL/min), (**b**) continuous desalination (*U* = 250 V, *V*_D_ = 20 μL/min, *V*_C_ = 2 μL/min), (**c**) unsuccessful operation (*U* = 50 V, *V*_D_ = 15 μL/min, *V*_C_ = 2 μL/min), and (**d**) desalination by accumulation (*U* = 250 V, *V*_D_ = 10 μL/min, *V*_C_ = 5 μL/min).

The time dependencies of the outlet concentrations typical for each category are plotted in Figure 2. The individual graphs in Figure 2 also contain insets capturing fluorescein signals obtained at the same desalination conditions. Figure 2a,b shows two examples of successful continuous desalination. The applied conditions were 200 V, 20 μL/min (flow rate of the diluate), and 5 μL/min (flow rate of the concentrate) in the first experiment, and 250 V, 20 μL/min (flow rate of the diluate), and 2 μL/min (flow rate of the concentrate) in the second one. The trends in the concentration-time profiles and the quality of desalination confirm the successful performance of continuous desalination/concentration. This mode of operation is characterized by an almost immediate decrease in KCl concentration in the diluate and a relatively slow increase in the concentrate concentration. Such a concentration dynamic is primarily dictated by the flow rates through particular channels. While a high diluate flow rate (20 μL/min) carries the desalted solution to the measuring electrodes within a few minutes, a small concentrate flow rate (5 μL/min) prolongs this time to about 15 min, as seen in the first experiment (Figure 2a). At smaller concentrate flow rates (2 μL/min), the concentrate concentration did not reach a steady value even after 30 min of operation (Figure 2b).

The fluorescein signal (the inset in Figure 2a) documents the main features of continuous desalination. While no fluorescence is detected in the desalination channel, high and evenly distributed fluorescence is excited in the concentration channel. The fluorescein detection only in the concentrate is a direct visual proof that charged molecules separate into this stream. This observation is consistent with the measured conductivities of both output streams. Interestingly, the fluorescein is almost undetectable in the feed stream. The signal coming from this channel is of much lower intensity than that in the concentrate. The step-change in the fluorescence intensity at the channel junction evidences the local fluorescein accumulation and, thus, the increase in its concentrate concentration. Another feature of continuous desalination is the seeming violation of the KCl balance. Since there is virtually no KCl in the diluate, all KCl entering the chip shall leave the chip as the concentrate (see, for example, Figure 2a). The KCl theoretical concentrate concentration is five times larger than that in the feed. This factor is given by the ratio of feed to concentrate flow rates predicting the concentrate concentration of 10 mM. However, the actual output concentration is approximately 4.5 mM. As shown later in the desalination by accumulation experiments, the system also accumulates ions in the space between the two cation-exchange membranes, mainly in the vicinity of the auxiliary membrane. The accumulation of ions in this space is a result of the membrane concentration polarization. The difference in the KCl amount fed into and withdrawn from the chip, thus, gives the KCl amount accumulated in the intermembrane channel. A similar situation occurs in Figure 2b, in which the theoretical concentrate KCl concentration should be 10 times larger than its concentration in the feed, that is, 20 mM. Although the concentration profiles show the system has not reached a steady value of the concentrate concentration, its value is only about 8 mM after 30 min of operation.

Figure 2c depicts the course of an experiment that was classified as an unsuccessful operation. The experimental conditions were 50 V, 10 μL/min (flow rate of the diluate), and 2 μL/min (flow rate of the concentrate). KCl concentration in the diluate almost does not differ from that in the feed. The concentration in the concentrate increases by about 50%, although the theoretical concentration factor for those conditions is 6. The unsuccessful operation is also visible from the fluorescein signal, which comes from both the diluate and concentrate channels. The main reason for this unsuccessful operation of the chip is the low voltage connected to the system. The ion-depleted zone cannot resist the convective flow and, thus, becomes leaky for ions.

Finally, the experiment in Figure 2d was categorized as desalination by accumulation. In this case, the conditions applied were 250 V, 10 μL/min (flow rate of the diluate), and 5 μL/min (flow rate of the concentrate). Large voltages and small diluate flow rates are characteristic of this mode of operation. The time-concentration profiles for both the diluate and concentrate showed the same trend, that is, the concentration decreased to low values (see Figure 2d). Surprisingly, the concentration in the diluate was even larger than that measured in the concentrate. The explanation for this behavior is based on the behavior of fluorescein under these conditions (the inset in Figure 2d). The fluorescein accumulates at the channel branching and cannot enter either the concentrate or diluate channel. The applied conditions make the ion-depleted zone grow out of the diluate channel into the main one. The outgrown depleted zone largely blocks the passage of most ionic species. Since the flow rate through the diluate channel is more significant than that through the concentrate channel, the diluate concentration is higher than the concentrate concentration. In this case, both output streams are virtually rid of ionic species that accumulate in the intermembrane region.

Desalination by accumulation is a typical arrangement in ICP-based systems removing charged species [41]. In those systems, however, the increase in the flow rate leads to (partial) destruction of the ion-depleted zone, which becomes leaky for ions. The increase in the flow rate in our system generates the concentrate stream without affecting the quality of the diluate.

### 2.4. Mapping the Types of Operation

The experiments described above represent the system’s possible types of operations uniquely dependent on the applied conditions. The dependence on the applied conditions can be summarized in the following way. There are two limiting cases given by the connected voltage. In one case, when the applied voltage is low, the ion-depleted zone is weak and cannot block the ionic species in their flow through it. This desalination was referred to as an unsuccessful operation. In the other case, when the connected voltage is high (and the flow rates are relatively low), the ion-depleted zone grows into the main channel, causing the desalination of both output streams. This operation was denoted as desalination by accumulation. The last observed case, called continuous desalination, occurs at medium voltages and flow rates. This case allows one to recover both the desalted water and preconcentrated solution continuously. To map the parametrical space and determine in what regime the microfluidic chip operates, we arranged the performed experiments in a table and assigned them the type of operation. This assignment is depicted in Table 2. Here, the columns contain the applied diluate and concentrate flow rates, and the rows contain the connected voltage. The columns are arranged in the order of increasing diluate flow rate and sub-arranged in the order of growing concentrate flow rate. The rows are arranged ascendingly according to the voltage. The capital letters C, X, and A stand for continuous desalination, unsuccessful operation, and desalination by accumulation, respectively. Qualitatively, the Table divides into three parts by two declining lines. The upper-right corner of the Table contains mainly unsuccessful operation, the bottom-left corner the desalination by accumulation, and the middle band the continuous desalination. This qualitative division of the Table agrees well with our observations described in Section 2.3. The unsuccessful operation occurs at low voltages and higher flow rates, the desalination by accumulation at high voltages and lower flow rates, and finally, the continuous desalination at medium voltages and medium flow rates.

### 2.5. Desalination Factor

**Figure 3:**
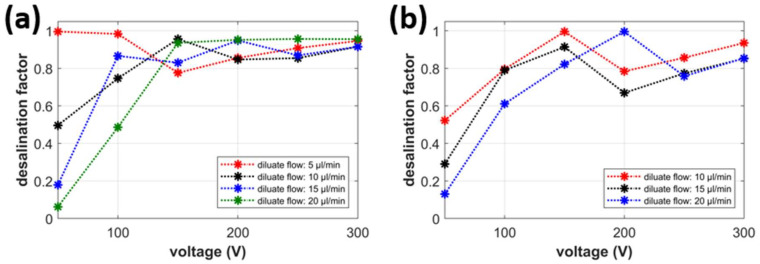
Desalination factor; (**a**) voltage dependence (*V*_C_ = 2 μL/min), (**b**) voltage dependence (*V*_C_ = 5 μL/min).

As described in Section 3.5, we calculated two parameters by quantitatively evaluating the degree of desalination/concentration. The first factor is the desalination factor *DF*, defined as the ratio of the difference between the inlet and diluate outlet concentrations and the inlet concentration (*DF* = (*c*_IN_ − *c*_D_)/c_IN_). If *c*_D_ approaches zero (the wanted state), then *DF* approaches 1, and the desalination is successful. If *c*_D_ is equal to *c*_IN_, then *DF* is equal to 0, and the desalination does not occur. The channel network works as a simple splitter of the feed solution. Figure 3a,b shows the dependence of *DF* on the connected voltage at various flow rates of the diluate at the flow rate of the concentrate equal to 2 and 5 μL/min, respectively. The values of *DF* were evaluated from the concentration values obtained at the end of the experiments.

The value of the desalination factor increases with the increasing voltage connected to the system for all tested flow rates through the diluate channel. These dependencies are not perfectly monotonous; however, the growth of *DF* toward 1 is evident. In several cases, the voltage increase worsens the quality of desalination by up to 20% (e.g., see the red curve in Figure 3b for 150 and 200 V). One possible explanation for this effect is that this voltage also drives the overlimiting electroconvection, which causes mixing of the ion-depleted zone with the solution bulk. The pressure-driven convection can then force the ions present in the ion-depleted zone into the diluate channel. Low values of the driving voltage (50 V) display only poor or minor desalination for the diluate flow rates larger than 5 μL/min. This is consistent with the previous results in which fluorescein leaked into the diluate channel at this voltage. The dependence of desalination on the flow rate through the concentrate channel does not seem significant. The results in Figure 3a,b are very similar, with *DF* values being between 80 and 100 percent in most of the experiments.

### 2.6. Concentration Factor

**Figure 4:**
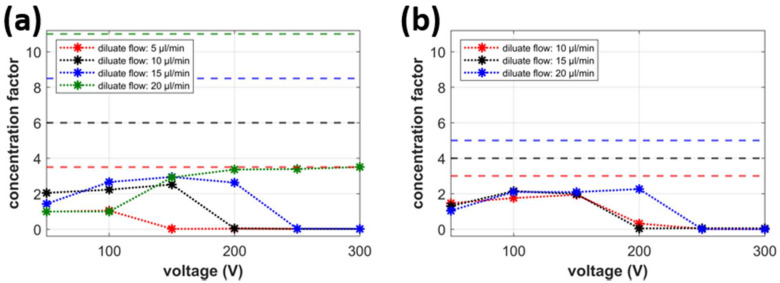
Concentration factor (solid lines with markers) and theoretical concentration factor (dashed lines); (**a**) voltage dependence (*V*_C_ = 2 μL/min), (**b**) voltage dependence (*V*_C_ = 5 μL/min).

The second evaluated factor is the concentration factor, defined as the ratio of the concentrate and feed concentrations (*CF* = *c*_C_/*c*_IN_). This factor quantifies how many times the solute is concentrated in the concentrate stream. This is valuable information in applications related to the preconcentration of (biological) reagents or waste to be processed. A *CF* value larger than 1 indicates a successful solute concentration. Its value equal to 1 means that the feed and concentrate concentrations are the same, which means that the chip works as a simple splitter. Figure 4a,b displays the dependence of *CF* on the connected voltage at various diluate flow rates at the flow rate of the concentrate equal to 2 and 5 μL/min, respectively. Figure 4 shows that the solute concentration does not take place at low voltages (50–100 V). The *CF* value is equal to one or very close to one for those low voltages. This observation is in agreement with the results plotted in Figure 4a,b showing almost no desalination of diluate under those conditions. The strength of the ion-depleted zone to act as an ionic filter increases with increasing connected voltage. The concentration factor between 2 and 3 is obtained for the voltages in the range of 100 and 200 V. However, the application of any larger voltage causes the *CF* factor to approach zero. This seemingly wrong result indicates the proceeding desalination by accumulation (see the *DF* in Figure 3 under these conditions). The ion-depleted zone grown into the main channel prevents the ions from entering the concentrate. The concentrate stream thus becomes desalinated and exhibits low concentrations of the KCl. This effect is more dominant for lower diluate flow rates.

The graphs in Figure 4 also show the theoretical concentration factor *CF*_T_ plotted as horizontal dashed lines. This theoretical factor represents a maximum possible concentration achievable in the chip and is given simply by the ratio of inlet and concentrate flow rates implying all ions in the feed solution separate into the concentrate and do not accumulate in the chip. Comparing the measured *CF* with the *CF*_T_ indicates that this is not the case in any case study. At low values of the connected voltage, there is an appreciable amount of ions leaving the chip with the diluate. At medium voltages, a noticeable amount of ions accumulate in the intermembrane region of the main channel and do not enter the concentrate. Complete removal of ions from both the diluate and the concentrate occurs at large voltages for which *CF* approaches 0. The desalination by accumulation, in turn, implies that the water recovery given as a ratio of diluate and concentrate flow rates is infinite (the system produces no concentrate).

### 2.7. Fluorescein Experiments

**Figure 5:**
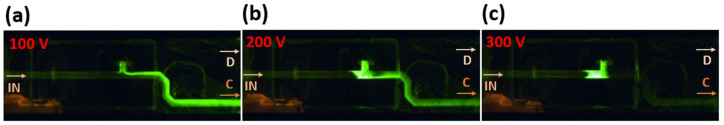
Visualization of desalination with the fluorescein solution for different values of inserted voltages at *V*_D_ = 10 μL/min, and *V*_C_ = 5 /min. (**a**) continuous desalination, (**b**) unsuccessful operation, (**c**) desalination by accumulation.

The previous results showed that complete continuous desalination of the processed solution occurs for a relatively large range of operational conditions. Interestingly, complete separation of the removed ions into the concentrate occurs for none of the applied conditions. This is documented by the values of the concentration factor being several times lower than the theoretical ones. The missing ions accumulate in the system. We performed several desalination experiments with KCl solution spiked with fluorescein to characterize the system’s ability to continuously concentrate ionic entities. Figure 5 depicts the fluorescein signal recorded after 30 minutes of desalination for voltages of 100, 200, and 300 V at constant diluate and concentrate flow rates of 10 and 5 µL/min, respectively. These operating conditions provide successful desalination or desalination by accumulation, as evidenced by Table 1. The fluorescein signal is qualitatively consistent with the results plotted in Figure 4b (red line). This line represents the concentration factor obtained for the same flow rates at different voltages. 

At 100 V (Figure 5a), the fluorescein continuously separates into the concentrate without accumulating at the ion-depleted zone. According to the intensity of fluorescence, fluorescein separation into concentrate is very efficient and approaches 100%. This is in quantitative disagreement with the measurement for KCl separation. The concentration factor around 1.8 compared to the theoretical value of 3 at 100 V means KCl also accumulates in the system. Its efficiency of separation into the concentrate reaches a value around 60% only. This result suggests the continuous removal of ions from the diluate is independent of the nature of ions, unlike their continuous separation into the concentrate. This statement is nicely documented by the results obtained at 200 V. The concentration factor essentially reaches zero, indicating complete accumulation of KCl in the system. However, the fluorescein signal is still high in the concentrate channel, although one can see fluorescein accumulation at the junction of the diluate and concentrate channel. Under the voltage of 300 V, both KCl and fluorescein accumulate entirely in the system. The accumulation is evidenced by the zero value of the concentration factor and no fluorescence in the concentrate channel. The fluorescein accumulates at the channel junction, where it creates a well-defined band of very intensive fluorescence.

## 3. Material and Methods

### 3.1. Material

The materials used for the fabrication of the microfluidic chip included 2 mm thick plexiglass plates (Zenit, Prague, Czech Republic), Tygon tubing (Watrex, Prague, Czech Republic), and Acrifix^®^ 1R0192 and 1S0117 (Zenit, Prague, Czech Republic). The functional membranes were heterogeneous cation-exchange membranes with the brand name Ralex CEM (MemBrain, Stráž pod Ralskem, Czech Republic). The chemicals used in this work included Tris-Acetate-EDTA (Sigma-Aldrich, Prague, Czech Republic), potassium chloride p. a. (PENTA, Prague, Czech Republic), and fluorescein (Fluka/Sigma Aldrich, 46955, Prague, Czech Republic).

### 3.2. Chip Design and Fabrication

**Figure 6:**
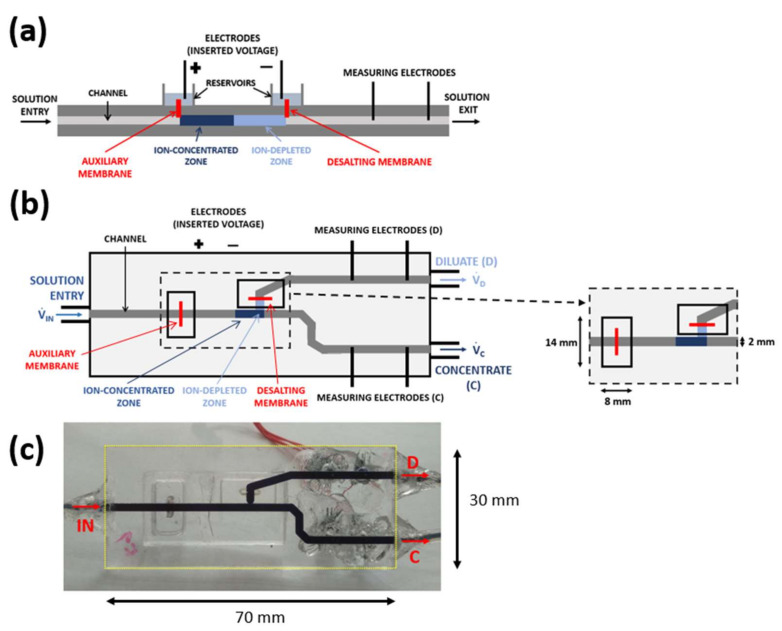
(**a**) Schematics of the microfluidic chip designed for continuous ICP-based desalination/preconcentration—side-view, (**b**) schematics of the microfluidic chip—top-view, (**c**) the picture of the completed microfluidic chip filled with a solution of trypan blue for an easier view of the channel network.

Figure 6a,b shows the schematics of our ICP-based desalination/concentration microfluidic system. The inlet channel splits into two channels, one for the desalted water solution (diluate, D) and one with the concentrated solution (concentrate, C). Two cation-exchange membranes are placed on top of the channel network (flush with the top channel wall) at the given locations. They separate the channels from the electrode reservoirs in which platinum electrodes for connecting the external voltage are placed. The CEM placed on top of the inlet channel is labeled as an auxiliary membrane. The other CEM located beyond the entrance into the diluate channel is referred to as a desalting membrane. 

The fabrication of the chip proceeded in the following steps. Gravos GV21 CNC milling machine (Prusa Research, Prague, Czech Republic) produced fluidic and other needed structures in two plexiglass plates (70 × 30 mm^2^). The upper and bottom plexiglass plates contained two rectangular openings to fit the CEMs and the channel network, respectively. All channels were 300 µm deep and 2000 µm wide. Low-viscosity one-component glue Acrifix 1S0117 bonded the structured plates together. Two small CEM pieces were inserted in the corresponding openings and left to swell in a KCl solution for 48 h. Acrifix 1R0192 sealed these membranes. This glue also fixed the electrode reservoirs and short pieces of Tygon tubing, serving as the inlet and outlets to the chip body. To monitor the desalination in real time, we integrated a pair of measuring electrodes into the diluate and concentrate channels. The electrodes measure the impedance of the flowing solutions, from which their concentration is evaluated through performed calibrations. The picture of the completed microfluidic chip is in Figure 6c.

The external DC field connected to the system generates ion-depleted and ion-concentrated regions at the desalting and auxiliary membrane, respectively. Since the desalting membrane is positioned 3 mm behind the entrance into the diluate channel, the ion-depleted zone mainly occupies the entrance zone of this channel. It therefore blocks ionic components from entering this channel. The feed solution at the channel bifurcation splits into a desalted solution flowing through the diluate channel and concentrated solution flowing through the concentration channel. The flow rate through individual channels is controlled independently with a syringe pump operating in a withdrawal mode.

### 3.3. Experimental Setup

**Figure 7:**
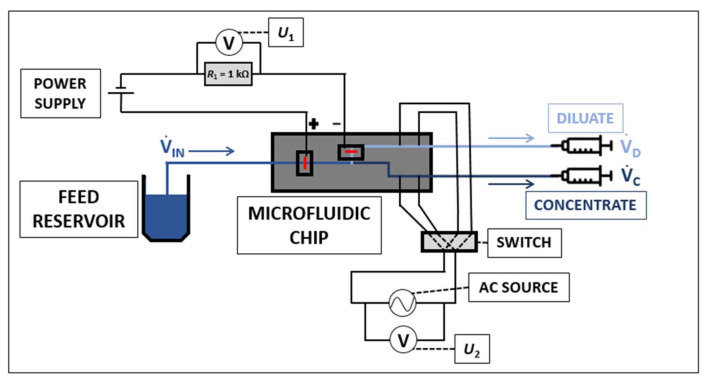
Schematics of the experimental setup used for desalination.

The schematic of the experimental setup is depicted in Figure 7. The feed solution flows from its reservoir into the main channel of the microfluidic chip. Here, it separates into a diluate and a concentrate solution. The flow is invoked by two syringe pumps working in the withdrawal mode (New Era Pump Systems, New York, USA), which control (set) the flow rates in the diluate and concentrate channel. The total flow rate of the feed solution is the sum of the diluate and concentrate flow rates. The electrode chambers are filled with a TAE buffer (Tris-Acetate-EDTA). TAE buffer has a large pH buffering capacity and prevents pH changes in the channels. Platinum wires are used as the electrodes for the connection of the external voltage. They are placed in the appropriate electrode chambers. The electric current *I*_1_ flowing through the circuit during the desalination is evaluated from the electric voltage *U*_1_ measured on a resistor of 1000 ohms connected in the circuit. This arrangement prevents any damage to the used measurement units in case of an electric shortcut. A power source Consort EV3150 connects the driving voltage on the system. A multimeter Agilent 34410A measures the electric voltage *U*_1_. Two pairs of platinum electrodes placed in the diluate and concentrate channels (measuring electrodes in Figure 6a,b) are alternatively connected to an AC source Keithley AC and DC Current Source 6221. This source imposes an AC signal with an amplitude of 10 µA and a frequency of 500 Hz to those electrodes. The response RMS voltage is measured on a digital multimeter Pro’sKit MT-1710, and the corresponding concentration of KCl is determined from calibration curves (see below).

We performed additional experiments with a fluorescein solution to visualize the process of desalination/concentration. A PixeLINK PL-D722CU camera (Rochester, NY, USA) acquired the pictures of the fluorescein removal from the desalted stream (its preconcentration). We used a Dark Reader Blue Transilluminator DR22A (Clare Chemical Research, Dolores, CO, USA) to excite and record the fluorescein fluorescence in the channels. The dark reader excites fluorescence by exposing the studied system to blue light. The emitted fluorescence passes through an orange plate and is recorded by the PixeLINK camera (Rochester, NY, USA).

### 3.4. Desalination Experiments

Both syringe pumps set to the required flow rates are switched on in the withdrawal mode at the beginning of the experiment. The initial concentrations of both the diluate and concentrate streams are measured. After 5 min, the DC power supply connects the chosen voltage to the system. The measurement of the passing electric current starts at the same time. We monitor the desalination course by measuring the concentration of the diluate and concentrate streams every 3 min. The driving voltage is turned off after 35 min from the commencement of the experiment. The channels are flushed thoroughly with a fresh KCl solution before the following experiment.

All desalination experiments were performed with 0.002 M KCl solutions. Fluorescein at the final concentration of 10–5 M added to 0.002M KCl solution was used in the additional experiments. We studied the effect of two input parameters on the desalination/concentration efficiency: the volumetric flow rates of the diluate and concentrate streams and the applied voltage. We tested the following driving voltages: 50, 100, 150, 200, 250, and 300 V. The following combinations of the volumetric flow rates written as the flow rate of the diluate/flow rate of the concentrate were applied: 5/2, 10/2, 15/2, 20/2, 10/5, 15/5, 20/5, and 20/10 μL/min.

### 3.5. Results Evaluation

The measured concentrations of the outlet streams as a function of time served to calculate two factors determining the desalination and concentration efficiency. The desalination factor, evaluated as *DF = (c*_IN_−*c*_D_*)/c*_IN_, where *c*_IN_ and *c*_D_ are concentrations of the feed solution and the diluate, respectively, characterizes the desalination quality achieved in the diluate stream. The *DF* values range between 0 and 1, where 0 means no desalination and 1 indicates 100% efficient separation. We defined a concentration factor as *CF = c*_C_*/c*_IN_, where *c*_IN_ and *c*_C_ are the concentrations of the feed solution and the concentrate, respectively, to quantify the salt concentration in the chip. *CF* is equal to 1 when no concentration occurs and is larger than 1 if the separation of salt ions is successful. The higher is its value, the more concentrated is the concentrate solution. The theoretical *CF* maximum for 100% separation efficiency is given by the ratio of the feed solution and the concentrate flow rates.

## 4. Conclusions

We developed a separation process based on concentration polarization of two cation-exchange membranes, where we separated both small ions and larger molecules from their water solution. We identified three regimes of operation set solely by the imposed conditions, namely, the flow rates and the connected voltage. Under optimal conditions, the system produced a desalted diluate rid of ions and concentrate. The results indicated that small ions always accumulate in the inter-membrane region. The desalination factor ranged from 80 to 100%, depending on the experimental conditions. The concentration factor and water recovery, which are experimentally predetermined by the ratio of the output flow rates, reached 0 and infinity, respectively, for the desalination by accumulation. The system is a versatile platform allowing on-demand desalination/concentration of small volumes of samples.

## Figures and Tables

**Table 1:** The calibration curves used to calculate the KCl concentration solution from the measured magnitude of the impedance.

Concentration Interval	(0; 0.2) mM	(0.2; 20) mM
Concentrate channel	*c*_C_ = 4355∙(1/*R*_2_)^2^ − 8.593∙(1/*R*_2_) + 0.004	*c*_C_ = 1.0982∙(1/*R*_2_) − 0.0009
Diluate channel	*c*_D_ = 3533∙(1/*R*_2_)^2^ − 6.913∙(1/*R*_2_) + 0.0033	*c*_D_ = 1.0010∙(1/*R*_2_) − 0.0008

**Table 2:** Mapping the types of operations: Each experiment is specified by the value of the inserted voltage (V) and by the combination of the outlet volume flows (written as *V*_D_⁄*V*_C_ in μL/min). The three types of operation are denoted as C—continuous desalination, X—unsuccessful operation, and A—desalination by accumulation.

		*V*_D_*/V*_C_ (μL/min)							
		5/2	10/2	10/5	15/2	15/5	20/2	20/5	20/10
***U* (V)**	**50**	C	X	C	X	X	X	X	X
	**100**	C	C	C	C	C	X	C	C
	**150**	A	C	C	C	C	C	C	C
	**200**	A	A	A	C	A	C	C	C
	**250**	A	A	A	A	A	C	A	C
	**300**	A	A	A	A	A	A	A	A

## Data Availability

Not applicable.
